# Internet addiction and mental health disorders in high school students in a Peruvian region: a cross-sectional study

**DOI:** 10.1186/s12888-023-04838-1

**Published:** 2023-06-07

**Authors:** Jean C. Perez-Oyola, Dionela M. Walter-Chavez, J. Pierre Zila-Velasque, César Johan Pereira-Victorio, Virgilio E. Failoc-Rojas, Víctor J. Vera-Ponce, Danai Valladares-Garrido, Mario J. Valladares-Garrido

**Affiliations:** 1 Faculty of Medicine, Universidad de San Martín de Porres, Chiclayo, Peru; 2 Universidad Nacional Daniel Alcides Carrion, Facultad de Medicina Humana, Pasco, Peru; 3 Red Latinoamericana de Medicina en La Altitud E Investigación (REDLAMTAI), Pasco, Peru; 4 School of Medicine, Universidad Continental, Lima, Peru; 5 Unidad de investigación para la generación y síntesis de evidencias en salud, Universidad San Ignacio de Loyola, Lima, Peru; 6 Instituto de Investigación en Ciencias Biomédicas, Universidad Ricardo Palma, Lima, 15039 Peru; 7 Universidad Tecnológica del Perú, Lima, 15046 Peru; 8 Escuela de Medicina, Universidad Cesar Vallejo, Piura, Peru; 9 Oficina de Epidemiología, Hospital Regional Lambayeque, Chiclayo, Peru

**Keywords:** Internet addiction disorder, Anxiety, Depression, Adolescent, Hispanic or Latino

## Abstract

**Objectives:**

To determine the association between internet addiction disorder (IAD) and anxiety and depressive symptomatology in high school students in two private schools in Chiclayo, Peru, during the COVID-19 pandemic.

**Materials and methods:**

Analytical cross-sectional investigation of 505 adolescents from two private schools. The dependent variables were anxiety and depressive symptomatology, measured with the Beck Adapted Depression Questionnaire (BDI-IIA) and the Beck Anxiety Inventory (BAI), respectively. The main independent variable was IAD, measured with the Internet Addiction Test instrument(IATI). Prevalence ratios (PR) and 95% confidence intervals (95%CI) were estimated.

**Results:**

The average age was 14.16 years and 54.9% were women. 22.2% and 3.2% presented mild and moderate IAD; respectively. 9.3% presented severe anxiety and 34.3% severe depressive symptomatology. In the simple regression, adolescents with mild, moderate and severe IAD presented 19% (PR = 1.19; 95%CI: 1.05–1.35), 25% (PR = 1.25; 95%CI: 1.02–1.53) and 53% (PR = 1.47; 95% CI: 1.47–1.60) higher prevalence of depressive symptomatology; however, this association was not maintained in the multiple model. Anxiety increased 196% in adolescents with severe IAD (PR = 2.96; 95%CI: 1.86–4.71).

**Conclusion:**

We found that 2, 1, and 3 out of 10 students presented IAD, depressive symptomatology, and anxiety, respectively. We did not find an association between IAD and depressive symptomatology, but we did find an association with anxiety. Among the factors associated with the development of depressive symptomatology were the male sex, the presence of eating disorders, subclinical insomnia, using devices for more than 2 h, and using the Internet for academic activities. About anxiety, the associated factors are the female sex, the presence of eating disorders, subclinical insomnia, and the use of the Internet as social interaction. We recommend implementing counseling programs in view of the imminent introduction of the Internet as a pillar in education.

## Introduction

In December 2019, the first case of unknown pneumonia was reported in China, later named by the World Health Organization (WHO) as coronavirus disease (COVID-19) [1]. More than 600 million confirmed cases and over 6 million deaths had been reported [2]. In Peru, containment measures were implemented to mitigate the increase in infections through social isolation and quarantines [3] due to its proven effects in reducing the number of infections and deaths [4]. However, these measures that keep people at home have conditioned the increased internet use through electronic devices (computers, cell phones, tablets or other devices with a screen) [5] that were established as the only means of communication, however, its excess leads to the consequent appearance of mental health disorders (anxiety and depression) [6–8], which occurs to a greater extent in adolescents due to their psychological vulnerability [9].

Globally, before the time of COVID-19, internet use was 57.0% [10], report that in the context of the pandemic it has increased up to 70.0% [6]. According to Peruvian statistics, 79.4% of the population connects to the Internet daily, while 76.8% correspond to the age group of 12 to 18 (high school students) [11]. Similar to what was found in Mexico (6 to 17 years old), with an intensity of use of 5 h a day [12]. Result supported by a meta-analysis carried out at the time of COVID-19 showed a range of exposure to a screen from 5 to 10 h per day [9] in adolescents. Turning this exposure time into a factor with a great impact on health [6], as the development of aggression [13], predict suicidal risk [14], greater psychopathology and more temperament difficulties [15] that have been greater in the time of confinement. In relation to the prevalence of internet addiction disorder (IAD), they have been reported from 3.5% and 6.2% in the pandemic context in European students [16], different from what was found in Asian high school students with 24.4% [17]. Concerning the prevalence of mental health disorders (anxiety and depression) in adolescents, the prevalence before the pandemic ranged between 11.6% and 12.9%, respectively [18, 19]. However, the pandemic's isolation has increased anxiety prevalence to 24.2% among adolescents aged 13 to 16 [20], similarly, and depression up to 16.0% [21]. Results are supported by a meta-analysis that reported a prevalence of 25.9% and 20.5%, in anxiety and depression, respectively [22] which was higher in older adolescents and girls.

Regarding the association between IAD and the development of mental health disorders in schoolchildren, a positive association has been found between both variables [23]. European and Asian studies show that the association was positive [24, 25], as well as other investigations that found that IAD is an influential factor for stress, depression, anxiety, and loneliness [26]. These results are supported by different meta-analyses carried out in the context of the COVID-19 pandemic [9, 27, 28]. However, the studies carried out in other contexts presented a series of limitations. One of them is a small sample; others did not conclusively demonstrate the association of interest since previous studies have not estimated association measures [29] and in the context of the pandemic, having focused only on the adult population [27, 30] and in schoolchildren from contexts other than Peruvian [31–33].

Focusing on an unexplored population such as secondary school students, in addition to the increase in the use of the Internet, gives an urgent need to intervene from education, since there is a risk that this situation can be maintained in adulthood [34]. Due to the above mentioned in this study, we have the objective of determining the association between IAD and the presence of mental health disorders in high school students from a Peruvian region.

## Methods

### Study design

We conducted an analytical cross-sectional study on schoolchildren from two private schools in Chiclayo, Peru, to evaluate whether IAD is associated with mental health disorders (depressive symptomatology and anxiety). Data collection was carried out during the period August-November 2021. In this period characterized by the closure of schools due to the second wave of the COVID-19 pandemic in Peru, schools provided remote education to schoolchildren to prevent the spread of SARS-CoV-2. Particularly in the department of Lambayeque, strict mandatory social distancing measures were maintained since it was severely affected by COVID-19 in the first wave of the pandemic [35].

### Population and simple

The study population consisted of 810 enrolled schoolchildren from two private schools in the first to the fifth year of secondary level of the academic year 2021 (*N*_1_ = 630 and *N*_2_ = 180). The two schools included in the study are in Chiclayo, an urban city in the coastal region of Peru.

We aimed to recruit a sample size of 408 participants with a 95% confidence level, 90% power, and a 5% margin of error. Based on the estimated prevalence rates of 42% in the unexposed group and 58% in the exposed group, we calculated the required sample size. We also considered a 10% rejection rate by parents and schools, which led to an adjusted sample size of 490 participants. Ultimately, we recruited a total of 505 students from both schools, ranging from the first to the fifth year of secondary level, using non-probabilistic snowball sampling. The minimum number of students recruited by this method was 28, while the maximum number was 251.

**Fig. 1:**
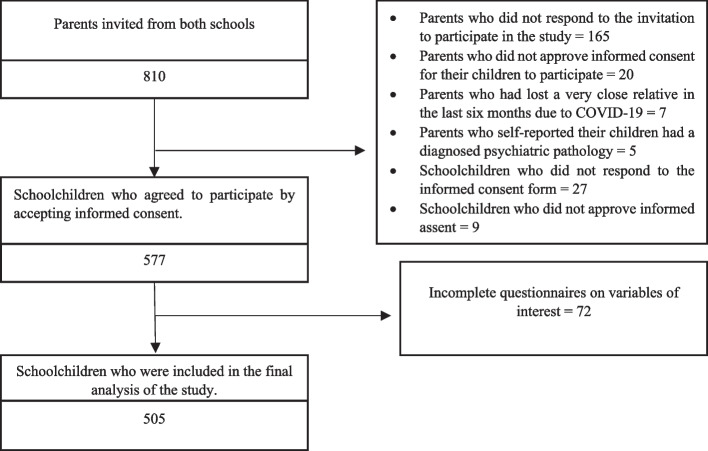
Participant selection flowchart

We included students who were enrolled in the 2021 academic year, regularly attended virtual classes, and had access to the internet at home for their educational activities. We excluded students whose parents reported that their children had a diagnosed psychiatric pathology, those who had lost a very close relative due to COVID-19 in the last 6 months (due to potential impact on students’ mental health and wellbeing), those who did not provide informed consent for their children to participate in the study, and schoolchildren who did not provide their assent to participate in the study. Finally, we obtained a sample of 505 students between the two private institutions (*n*_1_ = 381 y *n*_2_ = 124), which represented 62.3% of the population Fig. 1.

#### Variables

##### Outcome variable

The dependent variables were depressive symptomatology, defined with the Beck Depression Instrument when a score equal to or greater than 7 points is obtained, and anxiety, operationally defined as a score equal to or greater than 22 points obtained from the Beck Anxiety Instrument.

##### Variable exposure

The primary independent variable was Internet addiction, operationally defined as a score greater than 30 reported by the student through the responses to the Addiction Test Instrument.

##### Secondary independent variables

The covariates were personal, where age was investigated in years, sex (male, female), body mass index (underweight, normal, overweight, obese) after obtaining weight (in kilograms) and height (in meters). self-reported, insomnia (no, subclinical, moderate clinical, severe clinical insomnia), frequency of tobacco and alcohol use (sometime in their life, in the last year, in the last month), self-esteem (no, yes), eating behavior (no, yes), time on the internet device (less than 1 h, 1 to 2 h, 2 to 3 h, 3 to 4 h, more than 4 h), use of the internet for social interaction (no, yes), Internet use to play (no, yes), use of social networks (Facebook, Instagram, Twitter, WhatsApp, Snapchat, Tinder and Tiktok) and physical activity. Additionally, we explored educational variables: year of school study (first, second, third, fourth, fifth), self-report of having failed courses during their secondary school years (no, yes), and self-report of academic use of the Internet (no, yes).

### Instruments

Beck Depression Inventory Adapted (BDI-IIA): is an instrument that assesses the severity of depressive symptoms in people over 12 years of age in the last two weeks. It has 21 items, with Likert-type responses that are assigned a score from 0 (I don't feel sad) to 3 (I'm so sad and I feel bad). According to the cut-off points: a total score less than or equal to 6 is minimal depressive symptomatology; from 7 to 11, it is mild depressive symptomatology; 12 to 16, it is moderate depressive symptomatology; 17 to 63, it's severe depressive symptomatology [36]. The score adds up to 0 to 63 points at the end. Its validation in Peru was carried out in university students between 16 and 36 years old, where factorial analysis results were obtained with two factors (CFI = 0.97; IFI = 0.98; NFI = 0.83; NNFI = 0.97), internal consistency of 0.90 and stability very acceptable [37–39]. It has been used to assess mental health in the adolescent/general population during the pandemic in Latin America [40, 41].

Beck Anxiety Inventory (BAI): Its objective is to evaluate anxious symptoms in people over 12. It has 21 questions, with Likert-type answers that are given a score from 0 to 3: o = normal, 1 = mild, 2 = moderate, 3 = severe. With cut-off points, where: 0–21 = mild anxiety, 22–35 = moderate anxiety; more than 36 = severe anxiety [42]. Its validation in Peru was carried out on high school students from a Peruvian region, where Cronbach's alpha of 0.89 was obtained [43]. It has been used to assess mental health in the adolescent/general population during the pandemic in Latin America [44, 45].

Internet Addiction Test Instrument (IATI): the original instrument created by Young has 20 items and measures six factors: dominance, excessive use, neglect of work, anticipation, lack of control, and neglect of social life [46]. It uses a Likert-type scale from 0 to 5, depending on the behavior that describes "never", "rarely", "occasionally", "frequently", "very often," and "always". Validated in a sample of school adolescents from Lima-Peru [47], with a Cronbach's Alpha of 0.84. It has been used to assess TAI in the adolescent/general population during the pandemic in Latin America [48, 49].

Insomnia Severity Index (ISI): It is an instrument that consists of 7 questions and estimates the symptoms, worry and effects produced by insomnia. Each question has Likert-type responses scored from 0 to 4 (eg, 0 = no problem; 4 = very serious problem), giving a total score of 0 to 28 [50]. With cut-off points based on the original version, where: a score of 0–7 classifies it as not having insomnia; from 8–14, insomnia below the threshold; 15–21, clinical insomnia of moderate severity; 22–28, severe clinical insomnia [51]. It was translated into Spanish in a study with medical students showing a Cronbach's α = 0.82 [52].

Rosenberg Self-Esteem Scale(RSES): It consists of 10 questions, where 5 are directed in a positive way (items 1, 3, 4, 7 and 10) and 5 in a negative way (items 3, 5, 8, 9 and 10). It has 4 forms of response that are assigned a score: strongly disagree = 1, disagree = 2, agree = 3, and strongly agree = 4 [53]. The scores are from 10 to 40 points, a higher score indicates high self-esteem. For its validation in Peru, a population made up of 931 students between the ages of 11 and 18 belonging to public and private schools in Metropolitan Lima was used. The internal consistency was obtained by the coefficient H, giving values ​​ > 0.80 [54].

Eating Disorders Attitudes and Symptoms Self-Report Scale (EAT-26): It is an instrument that assesses the risk of presenting eating disorders in adolescents and adults of both sexes. Made up of 26 questions that are subdivided into 3 domains: Diet that corresponds to the items: 1, 6, 7, 10, 11, 12, 14, 16, 17, 22, 23, 24, 25, bulimia and preoccupation with food corresponds to the items: 3, 4, 9, 18, 21, 26, and oral control corresponds to the items: 2, 5, 8, 13, 15, 19, 20 [55]. The answers have 6 alternatives scored in a positive direction with the symptoms: Always = 3, Very often = 2, Often = 1, Sometimes = 0, Rarely = 0 and never = 0, only question 25 has an inverse direction. A score greater than 21 suggests a more exhaustive evaluation. Its validation in students of a Private University in Trujillo based on the Omega coefficient (w) showed a reliability of 0.90 [56].

Physical Activity Questionnaire for Adolescents (PAQ-A): Its objective is to assess the adolescent's physical activity in the last 7 days. It consists of 9 items that are scored on a five-point scale, where 1 = low level and 5 = high level of physical activity [57]. The first 8 questions are useful for scoring and question 9 allows you to know the reasons why you did not do physical activity that week [58]. The total score is the average value of the points obtained, the higher the score, the greater the physical activity [57]. It was validated in Peru through a study applied to 690 high school students in schools in the San Martin de Porres district who reported a Cronbach's alpha of 0.80 [59].

### Procedures

To ensure data quality control, we took several measures. Firstly, we designed the online questionnaire using the Google Forms platform. Secondly, we obtained authorization from school representatives, explained the research's purpose, and assured compliance with ethical principles for the participants. Thirdly, we conducted a pilot study with 50 students from the participating schools using previously validated standardized questionnaires to ensure an optimal data collection process. We administered the questionnaires in a quiet and comfortable environment and provided clear instructions to ensure consistency and accuracy. To achieve this, we had trained research assistants explain the survey's objectives to both parents and students prior to its administration.

To reach the student population, we asked the school representatives to distribute the online questionnaire to all students enrolled in the 2021 academic year via the online educational platform used during the COVID-19 pandemic. The survey was self-administered by the students themselves with no support from parents or teachers during its completion. The survey was disseminated during non-exam weeks and recess hours between virtual classes, and students had a maximum of 15 min to complete it.

### Analysis plan

We export the collected database from Google Forms in Excel format. Subsequently, we analyzed the data in the statistical program STATA v.17.0. We use descriptive statistics, according to the nature of the variables. We estimated absolute and relative frequencies for the categorical variables. In the numerical variables, we report the best measure of central tendency and dispersion, after evaluating the normal distribution.

In the bivariate analysis, we evaluated the association between mental health outcomes and IAD, as well as categorical covariates using the chi-square test, after evaluating the assumption of expected frequencies. In the case of numerical variables, the student's t test was used, after evaluating the assumption of normal distribution; otherwise, the Mann Whitney U test was useful.

To investigate the association between mental health disorders and IAD and the rest of the covariates of interest, we estimated prevalence ratios (PR) and 95% confidence intervals using generalized linear models (GLM), Poisson distribution family, function of log link and robust variance, in the simple and multiple regression analysis. In the final multiplex model, we used the covariates as adjustment for the association of interest (IAD and mental health disorders). Collinearity was evaluated between the confounding variables included in the final multiple model.

### Ethical aspects

This research work was reviewed and approved by the research ethics committee of the Universidad de San Martin de Porres Official letter No. 391–2021).The confidentiality of the information provided by the students was guaranteed, using anonymized database. Informed consent was requested from the parents of the minors surveyed, informing the purpose of the study and not disclosing any personal data. Additionally, informed assent was requested from schoolchildren whose parents authorized their participation.

We did not provide information on depression or internet addiction to schoolchildren, parents, or teachers after data collection to avoid any potential harm or stigmatization. However, we offered the school administration and parents the opportunity to receive the study results and participate in information sessions on the topic.

## Results

### General characteristics of schoolchildren

Of 505 adolescents evaluated, the mean age was 14.16 years, 54.9% were female, 21.8% were in their first year of studies, and 30.9% reported having failed a course. 28.7% and 1.6% reported having consumed alcohol and tobacco at some time in their lives; respectively. 17.4% had an eating disorder, and 3.6% presented moderate clinical insomnia. 48.7% reported using devices for more than 4 h a day to connect to the Internet. 47.9% and 27.9% mentioned using the Internet for academic purposes and social interaction; respectively. Regarding the use of social networks, the majority use Facebook (67.5%) and Instagram (55.6%); 22.2% (95% CI = 18.6%—26.1%) and 3.2% (95% CI = 1.9%—7.6%) presented mild and moderate IAD, respectively Table 1.

**Table 1:** Characteristics of participants (*n* = 505)

Characteristics	N (%)	95% CI
Age (years)^a^	14.16 ± 1.45	
Sex		
Male	228 (45.2)	
Female	277 (54.9)	
Year of studies		
First	110 (21.8)	
Second	106 (21.0)	
Third	97 (19.2)	
Fourth	100 (19.8)	
Fifth	92 (18.2)	
BMI (categorized)		
Underwight	35 (7.0)	
Normal	322 (64.1)	
Overweight	101 (20.1)	
Obese	44 (8.8)	
Alcohol consumption^b^		
Every in your life	145 (28.7)	
In the last year	93 (26.1)	
In the last month	46 (13.2)	
Tobacco use^b^		
Ever in your life	8 (1.6)	
In the last year	6 (1.9)	
In the last month	4 (1.2)	
Failing courses		
No	347 (69.1)	
Yes	155 (30.9)	
Self-esteem^a^	22.47 ± 3.56	
Physical activity^c^	1.89 (1.58–2.31)	
Eating Disorder		
No	417 (82.6)	
Yes	88 (17.4)	
Insomnia		
Absence of clinical insomnia	314 (62.2)	
Subclinical insomnia	169 (33.5)	
Moderate clinical insomnia	18 (3.6)	
Severe clinical insomnia	4 (0.8)	
Use of devices to connect to the Internet (hours)		
< 1 h	30 (5.9)	
1 to 2 h	73 (14.5)	
2 to 3 h	86 (17.0)	
3 to 4 h	70 (13.9)	
> 4 h	246 (48.7)	
Internet use for gaming		
No	263 (52.1)	
Yes	242 (47.9)	
Use of social networks		
No	364 (72.1)	
Yes	141 (27.9)	
Internet use for gaming		
No	393 (77.8)	
Yes	112 (22.2)	
Use of social networks^b^		
Facebook	341 (67.5)	
Instagram	280 (55.6)	
Twitter	130 (25.8)	
Whatsapp	363 (72.0)	
Snapchat	64 (12.7)	
Tinder	13 (2.6)	
Tiktok	279 (55.4)	
Internet addiction disorder		
Normal	374 (74.1)	69.9—77.2
Mild	112 (22.2)	18.6—26.1
Moderate	16 (3.2)	1.9—7.6
Severe	3 (0.6)	0.1—3.3
Anxiety		
Mild	365 (72.3)	68.8—75.0
Moderate	93 (18.4)	15.9—21.5
Severe	47 (9.3)	6.4—14.1
Depression		
Minimal	158 (31.3)	27.9—34.7
Mild	150 (29.7)	26.2—33.2
Moderate	24 (4.8)	2.9—7.1
Severe	173 (34.3)	30.9—37.7

^a^Mean ± standard deviation

^b^The variable has multiple answers

^c^Median (25th-75th percentiles)

Some variables do not up to 100% due to rounding

### Mental health disorders-instrument PHQ-9 and GAD-7

Eighteen-point four percent (18.4%, 95% CI = 15.9%—21.5%) and 9.3% (95% CI = 6.4%—14.1%) had moderate and severe anxiety, respectively. While 34.8% (95% CI = 30.9%—37.7%) and 29.7% (26.2%—33.2%) presented severe and mild depressive symptomatology, respectively Table 1.

### Association between IAD and mental health disorders, in bivariate analysis

Adolescents with severe and moderate IAD presented 34.8% (100% vs. 65.2%; *p* = 0.032) and 16.1% (81.3% vs. 65.2%; *p* = 0.032) higher frequency of depressive symptomatology, respectively, compared to adolescents who did not present IAD. The rest of the variables that were associated with depressive symptomatology were sex (*p* = 0.015), alcohol consumption (*p* = 0.028), eating disorder (*p* < 0.001), insomnia (*p* < 0.001), academic use of the Internet (*p* = 0.002) and use of the Internet to play (*p* = 0.011).

The frequency of anxiety was 7% (100% vs. 24.3%) and 75.7% (31.3% vs. 24.3%) higher in adolescents with moderate and severe IAD; respectively, compared to adolescents who did not present IAD. Additionally, sex (*p* = 0.002), eating disorder (*p* = 0.005), insomnia (*p* = 0.001) and use of the Internet to interact socially (*p* = 0.048) were associated with depressive symptomatology in the evaluated adolescents Table 2.

**Table 2:** Characteristics associated with depression and anxiety in bivariate analysis

Variables	***Depression***		p*	***Anxiety***		p*
	No (*n* = 158)	Yes (*n* = 347)		No (*n* = 365)	Yes (*n* = 140)	
	n(%)	n(%)		n(%)	n(%)	
Age (years)^**,a^	14.13 ± 1.32	14.18 ± 1.51	0.709	14.16 ± 1.38	14.16 ± 1.63	0.987
Sex			**0.015**			**0.002**
Male	84 (36.8)	144 (63.2)		180 (79.0)	48 (21.1)	
Female	74 (26.7)	203 (73.3)		185 (66.8)	92 (33.2)	
BMI (categorized)			0.858			0.839
Underweight	13 (37.1)	22 (62.9)		24 (68.6)	11 (31.4)	
Normal	97 (30.1)	225 (69.9)		236 (73.3)	86 (26.7)	
Overweight	32 (31.7)	69 (68.3)		70 (69.3)	31 (30.7)	
Obese	14 (31.8)	30 (68.2)		32 (72.7)	12 (27.3)	
Alcohol consumption			**0.028**			0.053
No	123 (34.2)	237 (65.8)		269 (74.7)	91 (25.3)	
Yes	35 (24.1)	110 (75.9)		96 (66.2)	49 (33.8)	
Tobacco use			0.248			0.156
No	157 (31.6)	340 (68.4)		361 (72.6)	136 (27.4)	
Yes	1 (12.5)	7 (87.5)		4 (50.0)	4 (50.0)	
Failing courses			0.431			0.764
No	113 (32.6)	234 (67.4)		253 (72.9)	94 (27.1)	
Yes	45 (29.0)	110 (71.0)		111 (71.6)	44 (28.4)	
Self-esteem	22.52 ± 3.26	22.44 ± 3.69	0.798	22.3 ± 3.47	22.89 ± 3.75	0.100
Physical activity	1.92 (1.56–2.31)	1.89 (1.58–2.31)	0.946	1.89 (1.61–2.31)	1.89 (1.5–2.28)	0.711
Eating Disorder			** < 0.001**			**0.005**
No	146 (35.0)	271 (65.0)		312 (74.8)	105 (25.2)	
Yes	12 (13.6)	76 (86.4)		53 (60.2)	35 (39.8)	
Insomnia			** < 0.001**			**0.001**
Clinical insomnia absent	123 (39.2)	191 (60.8)		246 (78.3)	68 (21.7)	
Subclinical insomnia	31 (18.3)	138 (81.7)		107 (63.3)	62 (36.7)	
Moderate clinical insomnia	3 (16.7)	15 (83.3)		9 (50.0)	9 (50.0)	
Severe clinical insomnia	1 (25.0)	3 (75.0)		3 (75.0)	1 (25.0)	
Use of devices to connect to the Internet (hours)			0.156			0.364
< 1 h	15 (50.0)	15 (50.0)		25 (83.3)	5 (16.7)	
1 to 2 h	24 (32.9)	49 (67.1)		52 (71.2)	21 (28.8)	
2 to 3 h	27 (31.4)	59 (68.6)		64 (74.4)	22 (25.6)	
3 to 4 h	17 (24.3)	53 (75.7)		45 (64.3)	25 (35.7)	
> 4 h	75 (30.5)	171 (69.5)		179 (72.8)	67 (27.2)	
Academic internet use			**0.002**			0.311
No	66 (25.1)	197 (74.9)		185 (70.3)	78 (29.7)	
Yes	92 (38.0)	150 (62.0)		180 (74.4)	62 (25.6)	
Internet use for social interaction			0.379			**0.048**
No	118 (32.4)	246 (67.6)		272 (74.7)	92 (25.3)	
Yes	40 (28.4)	101 (71.6)		93 (66.0)	48 (34.0)	
Internet use for gaming			**0.011**			0.466
No	134 (34.1)	259 (65.9)		281 (71.5)	112 (28.5)	
Yes	24 (21.4)	88 (78.6)		84 (75.0)	28 (25.0)	
Internet addiction disorder			**0.032**			**0.002**
Normal	130 (34.8)	244 (65.2)		283 (75.7)	91 (24.3)	
Mild	25 (22.3)	87 (77.7)		71 (63.4)	41 (36.6)	
Moderate	3 (18.8)	13 (81.3)		11 (68.8)	5 (31.3)	
Severe	0 (0.0)	3 (100.0)		0 (0.0)	3 (100.0)	

^*^ *p*-value of categorical variables calculated with the Chi-Square test

^**^ *p*-value of categorical—numerical variables calculated with the U-test (Mann–Whitney)

^a^Mean ± standard deviation

### IAD and other factors associated with mental health disorders, in simple and multiple regression analysis

In the simple regression analysis, we observed that the prevalence of depressive symptomatology increased 19% (PR = 1.19; 95%CI: 1.05–1.35), 25% (PR = 1.25; 95%CI: 1.02–1.53) and 53% (PR = 1.47 95% CI: 1.47–1.60) in adolescents with mild, moderate, and severe IAD; respectively. However, in the multiple model, the association was not found.

The factors associated with a higher prevalence of depressive symptomatology were male gender (PR = 1.15; 95%CI: 1.08–1.23), consuming alcohol (PR = 1.07; 95%CI: 1.01–1.13), having an eating disorder (PR = 1.25; 95%CI: 1.08–1.44), having subclinical insomnia (PR = 1.28; 95%CI: 1.12–1.46), using devices for 2 to 3 h (PR = 1.26; 95%CI: 1.02–1.56), 3 to 4 h (PR = 1.50; 95%CI:1.14–1.99) and more than 4 h (PR = 1.33; 95%CI: 1.11–1.60); and use the Internet for academic activities (RP = 0.85; 95% CI: 0.74–0.98) Table 3.

**Table 3:** Factors associated with depression and anxiety, in simple and multiple regression analysis

**Characteristics**	***Depression***						***Anxiety***					
	**Simple regression**			**Multiple regression**			**Simple regression**			**Multiple regression**		
	**PR**	**CI 95%**	***p*** *	**PR**	**CI 95%**	***p*** *	**PR**	**CI 95%**	***p*** *	**PR**	**CI 95%**	***p*** *
Age (years)	1.01	0.98–1.03	0.571	0.99	0.95–1.03	0.588	1.00	0.92–1.08	0.982	0.98	0.92–1.04	0.533
Sex												
Male	Ref			Ref			Ref			Ref		
Female	1.16	1.12–1.20	** < 0.001**	1.15	1.08–1-23	** < 0.001**	1.58	1.27–1.95	** < 0.001**	1.40	1.17–1.67	** < 0.001**
BMI(categorized)												
Underweight	Ref			Ref			Ref			Ref		
Normal	1.11	0.90–1.37	0.325	1.09	0.91–1.30	0.361	0.85	0.54–1.33	0.479	0.78	0.54–1.12	0.176
Overweight	1.09	0.84–1.40	0.523	1.09	0.85–1.40	0.488	0.98	0.63–1.52	0.916	0.88	0.57–1.36	0.566
Obese	1.08	0.81–1.45	0.582	0.98	0.72–1.35	0.920	0.87	0.51–1.47	0.598	0.82	0.40–1.69	0.596
Alcohol consumption												
No	Ref			Ref			Ref			Ref		
Yes	1.15	1.08–1.23	** < 0.001**	1.07	1.01–1.13	**0.025**	1.34	1.02–1.74	**0.003**	1.09	0.88–1.34	0.424
Tobacco use												
No	Ref			Ref			Ref			Ref		
Yes	1.28	1.05–1.56	**0.014**	1.13	0.85–1.50	0.411	1.83	0.87–3.82	0.110	1.57	0.71–3.46	0.261
Failing courses												
No	Ref			Ref			Ref			Ref		
Yes	1.05	0.98–1.13	0.166	1.03	0.91–1.16	0.687	1.05	0.78–1.41	0.759	1.04	0.87–1.23	0.697
Self-esteem	1.00	0.98–1.01	0.758	1.00	0.99–1.01	0.985	1.03	1.00–1.07	**0.045**	1.04	1.00–1.07	0.063
Physical activity	1.00	0.90–1.11	0.992	1.02	0.93–1.12	0.687	0.92	0.74–1.15	0.472	1.00	0.80–1.25	0.993
Eating Disorder												
No	Ref			Ref			Ref			Ref		
Yes	1.33	1.19–1.48	** < 0.001**	1.25	1.08–1.44	**0.003**	1.58	1.35–1.85	** < 0.001**	1.37	1.01–1.87	**0.046**
Insomnia												
Clinical insomnia absent	Ref			Ref			Ref			Ref		
Subclinical insomnia	1.34	1.18–1-52	** < 0.001**	1.28	1.12–1.46	** < 0.001**	1.69	1.22–2.36	**0.002**	1.42	1.01–2.01	**0.049**
Moderate clinical insomnia	1.37	1.17–1.60	** < 0.001**	1.18	0.89–1.57	0.251	2.31	1.76–3.04	** < 0.001**	1.44	0.97–2.13	0.073
Severe clinical insomnia	1.23	0.81–1.88	0.332	1.04	0.62–1.74	0.882	1.15	0.18–7.24	0.878	0.69	0.07–6.87	0.750
Use of devices to connect to the Internet (hours)												
< 1 h	Ref			Ref			Ref			Ref		
1 to 2 h	1.34	1.12–1.61	**0.002**	1.25	0.99–1.57	0.062	1.73	0.66–4.53	0.267	1.44	0.46–4.51	0.531
2 to 3 h	1.37	1.21–1.56	** < 0.001**	1.26	1.02–1.56	**0.033**	1.53	0.57–4.14	0.397	1.52	0.50–4.68	0.463
3 to 4 h	1.51	1.20–1.91	** < 0.001**	1.50	1.14–1.99	**0.004**	2.14	0.85–5.42	0.108	1.86	0.70–4.94	0.213
> 4 h	1.39	1.24–1.56	** < 0.001**	1.33	1.11–1.60	**0.002**	1.63	0.84–3.18	0.149	1.29	0.70–2.40	0.416
Academic internet use												
No	Ref			Ref			Ref			Ref		
Yes	0.83	0.70–0.97	**0.024**	0.85	0.74–0.98	**0.022**	0.86	0.66–1.14	0.295	1.10	0.88–1.39	0.405
Internet use for social interaction												
No	Ref			Ref			Ref			Ref		
Yes	1.06	0.96–1.17	0.243	0.96	0.80–1.15	0.661	1.35	1.01–1.80	**0.043**	1.31	1.07–1.61	**0.010**
Internet use for gaming												
No	Ref			Ref			Ref			Ref		
Yes	1.19	1.02–1.39	**0.024**	1.13	0.98–1.31	0.094	0.88	0.60–1.27	0.490	0.96	0.73–1.26	0.764
Internet addiction disorder												
Normal	Ref			Ref			Ref			Ref		
Mild	1.19	1.05–1.35	**0.006**	0.98	0.86–1.10	0.696	1.50	1.08–2.09	**0.015**	1.24	0.75–2.04	0.410
Moderate	1.25	1.02–1.53	**0.034**	0.95	0.82–1.09	0.455	1.28	0.45–3.66	0.639	0.81	0.28–2.31	0.690
Severe	1.53	1.47–1.60	** < 0.001**	1.08	0.96–1.22	0.201	4.11	3.35–5.05	** < 0.001**	2.96	1.86–4.71	** < 0.001**

^*^ *p*-values obtained with Generalized Linear Models (GLM), Poisson family, log-link function, and robust variance

Regarding anxiety, we found that adolescents with mild, moderate and severe IAD increased 50% (PR = 1.50, 95%CI: 1.08–2.09) and 311% (PR = 4.11; 95%CI: 3.35–5.05), the prevalence of anxiety, in the simple model. In the multiple model, only the association was maintained in severe IAD, since adolescents with this condition present a 196% higher prevalence of anxiety (PR = 2.96; 95%CI: 1.86–4.71); compared to those who do not have IAD. Female adolescents (PR = 1.42; 95%CI: 1.01–2.01), those who had an eating disorder (PR = 1.37; 95%CI: 1.01–1.87), subclinical insomnia (PR = 1.42; 95%CI: 1.01- 2.01) and the use of the Internet as a social interaction (PR = 1.31; 95% CI: 1.07–1.61) is associated with a higher prevalence of anxiety Table 3.

## Discussion

### Main findings

#### Prevalence of Internet Addiction Disorder (IAD)

We found that almost a third of the schoolchildren presented some IAD (25.9%), and the most frequent was mild IAD (22.2%). When compared to studies in non-COVID times, it is similar to that reported by Chi X. et al. in China, where he studied 532 adolescents, and a 22.6% prevalence was obtained in IAD [60]. Thabel J et al. found that 43.9% of Tunisian adolescents presented IAD [61]. However, it differs from what was found by Seyrek S. et al. who studied 468 schoolchildren, where it was found that 1.6% of the students presented IAD [24]. In Peru, Rosana Lluén, evaluated 800 students from the 1st to the 5th year of secondary school in five national schools, finding that 8.5% suffer from IAD, a risk of IAD was found in 25.7% [62] and another study carried out in our country found that 52.3% of the students surveyed in 4th and 5th grade of secondary school presented a medium level of the IAD [63].

While in studies during the COVID-19 pandemic, what was evidenced by Min-Pei Li is similar. in his research carried out in Taiwan with 1060 students, where he found that the prevalence of IAD was 24.4% [64]. Result similar to that evidenced in Peru with a prevalence of 21.4% of IAD in university students [65]. However, it differs from what was reported in Mexico, showing that 62.7% had IAD the general population [66], similarly in Ecuador with a prevalence of 51.0%, having university students as a population [67]. The prevalence of IAD is due to the fact that, during the COVID-19 pandemic, the internet became an essential tool that has maintained remote communication between people through social networks, videoconferences and video games; increasing the number of daily hours on the internet, causing the excessive use of these resources to lead to IAD in adolescents [68].

### Prevalence of mental health disorders

We found that almost 7 out of 10 schoolchildren presented depressive symptoms (68.8%) and severe depressive symptomatology (34.3%). Results in studies in times of the COVID-19 pandemic were lower than those found by Manar Al-Azzam et al. in high school students in Jordan, where 72.4% had mild to severe depressive symptomatology [69]. Also, it is similar to what was evidenced by Zeng Zhang et al., who collected data in three secondary schools in a Chinese province, where they found that the prevalence of mild to severe depressive symptoms was 52.4% [70]. However, it differs from the study by Peng Xiaodan et al. who estimated that 16.3% of the students had depressive symptomatology using the Patient Health questionnaire (PHQ-9) [71]. The high prevalence of depressive symptomatology, mainly severe depressive symptomatology, could be explained by the change from social life to isolation due to the COVID-19 pandemic, since the sudden change in lifestyles, and especially the impediment to go out and interact with For other people, the fear of getting sick or losing a family member contributes to the development of depressive symptoms in adolescents, even more so in those who are more psychologically vulnerable. This is corroborated because many meta-analyses suggest that the prevalence in children under 13 years of age is 2.8%, and increases to 5.7% in adolescents and young adults [72].

In addition, we found that almost 3 out of 10 schoolchildren presented anxiety, and 27.7% had moderate-severe anxiety. When comparing it with studies in non-COVID times, we found that it is similar to that reported by Ospina et al. who enrolled 538 schoolchildren between the ages of 10–17, where 28.3% presented symptoms suggestive of anxiety [73]. However, it is less than what was found by Thabel J. et al., who studied 253 adolescents, concluding that anxiety is present in 65.8% of adolescents [61]. While in studies in times of the COVID pandemic, our findings are similar to what was described by Zeng Zhang et al., where they found that the prevalence of mild to severe anxiety symptoms was 31.4% in Chinese school adolescents [70]. Finally, our result is supported by the evidence according to the United Nations Children's Fund (UNICEF) for Latin American adolescents with a prevalence of anxiety of 27.0% 74. However, it differs from the study carried out by Peng Xiaodan et al., who used the Generalized Anxiety Disorder Questionnaire (GAD-7), in a total of 39,751 Chinese school students, obtaining that the prevalence of anxiety symptoms is 10.3% [71]. Different situations can explain the prevalence of anxiety in our study: one of them is the age of the students, the average age being 14.16 years, at this age the prevalence of anxiety is higher, because they are in a transition from childhood to adult life. The presence of anxiety would also be produced by job uncertainty and fear of getting sick that their parents probably present, which is captured by adolescents, according to what was reported by UNICEF, 30.0% of adolescents are emotionally influenced by their economic situation, generating anxiety [74]. Likewise, the level of anxiety could be caused by remote or virtual education imposed as a measure to reduce the number of infections by COVID-19, which explains up to 30.0% of the presence of anxiety [75] and other pathologies (stress) [76].

### Internet addiction and mental health outcomes

Although in the multiple models there was no evidence of an association between IAD and depressive symptomatology, in the simple model we found that schoolchildren with mild, moderate, and severe IAD had a 19.0%, 25.0%, and 53.0% higher prevalence of depressive symptomatology, respectively. This result is supported by a study carried out in times of the COVID-19 pandemic, which also found no association of IAD with depressive symptomatology in the logistic regression model, despite the fact that the proportion of schoolchildren with depressive symptomatology was higher in those with IAD [64]. However, it differs from another pre-pandemic Peruvian study that demonstrated a statistically significant association between IAD and depressive symptomatology (PR: 2.17; 95% CI: 1.84 – 2.55, *p* = 0.001) [77]. It is also opposite to what was found by Seyrek S. et al. who found a positive correlation between IAT and depression in Turkish adolescents [24]. Ostovar S. et al. In his research carried out in Iran, he found that adolescents with higher levels of IAD had higher levels of depression [25]. Our association between IAD and depressive symptomatology could be explained due to the pandemic context that caused isolation and the transition to virtual education, which produced a greater use of the Internet and an increase in depressive symptoms, therefore, for some people, excessive use of the Internet predisposes to the development of depressive symptoms [78].

Additionally, in the multiple regression analysis, the covariates that resulted statistically significant were male sex, presence of eating disorders, subclinical insomnia, using devices for more than 2 h, and using the Internet for academic activities. These covariates may explain part of the association, as it is possible that those who spend more time on the Internet may also have more academic demands, experience more sleep disturbances, or have more difficulties regulating their eating habits. However, other underlying mechanisms, such as social isolation or negative online interactions, may also contribute to the association between Internet use and depressive symptoms.

Regarding anxiety, it was evidenced that the prevalence of anxiety increased by 196% in schoolchildren with severe IAD; compared to those who did not have IAD. This is contrary to what was described in an investigation carried out in times of the COVID-19 pandemic, where it was found that the higher the IAIT score, the lower the level of anxiety was obtained in medical students in Malaysia [79]. While studies carried out before the COVID-19 pandemic, as described by Ostovar S. et al. in their research with 1052 adolescents, found that IAD is significantly and positively related to an increase in anxiety, concluding that adolescents with higher levels of IAD had higher levels of anxiety [25]. Seyrek S. et al. found a positive correlation between the Young Internet Addiction Scale (YIAS) total score and the Beck Anxiety Inventory (BAI) total score [24]. Our association could be explained by the fact that TAI does not generate interpersonal relationships that behave as a measure of resilience that reduces the development of anxiety symptoms [80]. These types of personal relationships are transcendental for avoiding negative mental health symptoms in schoolchildren, however, during the COVID-19 pandemic, these relationships have not been generated due to forced isolation.

### Factors associated with depressive symptomatology

The greater the number of hours of use of devices to connect to the Internet, the greater the prevalence of depressive symptomatology. This is similar to what was reported by Ozlem F. et al. in times of the COVID-19 pandemic, where it found that adolescents who used the Internet for 3 h or more per day were more likely to have psychosocial problems, such as depressive symptomatology, compared to those who used the Internet less than 2 h per day [68]. It also agrees with what was described by Boonvisudhi T. et.al. who found that using the Internet for more than five hours a day was associated with a higher probability of depression [81]. However, it differs from what was found by Pérez T. et.al. that had secondary and high school students as a population, where the symptoms of depression were not correlated with the time spent on the Internet [82]. Our association could be explained because the COVID-19 pandemic led to an increase in the use of the Internet through devices to carry out different educational and social activities, substituting all kinds of face-to-face interaction for what would cause depression [83].

Schoolchildren who reported using the Internet for academic purposes reduced the prevalence of depressive symptomatology by 15.0%. This association was not found in any other similar research work. Our association could be explained because adolescents with a stressful academic load, such as exams have high levels of anxiety, so completing these types of tasks suppresses the development of depressive symptoms and at the same time obtains greater satisfaction that prevents this pathology [84].

Schoolchildren who had consumed alcohol at some time had a 7 times higher prevalence of depressive symptomatology. This is similar to what was reported in a study carried out in Mexico, where they estimated an association between alcohol consumption associated with depression in adolescents [85]. However, it differs from what was found by Estela S. Et. to the. in 678 Peruvian students, where no association was found between alcohol consumption and depressive symptomatology [77], like other research in Japan [86], and Italy [87]. Our association is because alcohol acts as a powerful nervous system depressant, which through small doses causes the false sensation of improvement in the symptoms of sadness, anxiety, and negative thoughts. However, its excessive consumption leads to the development of the depressant effects of alcohol through its connection with changes in the mesocorticolimbic, opioid and glutaminergic circuits [88] that produces changes in neurotransmission and the endocrinological system that, if maintained, will produce an increasing physical and mental deterioration, reaching to significantly affect the life of the person and the development of depressive symptoms [89].

Having an eating disorder increases the prevalence of depressive symptomatology by 25.0%. This is similar to what was reported by Carmiña M. et al. in Bolivia, who found a positive correlation between eating disorders and depressive symptomatology [90]. The proven relationship between eating disorders and depressive symptomatology could explain our association. It has been shown that the presence of bulimic symptoms contributes to the symptoms of depression, since these symptoms can provoke feelings of shame and guilt leading to the deterioration of the person [91] demonstrating that the presence of some eating disorder is associated with a four-fold increase in the risk of developing depression [92]. However, this controversy remains, so our results add to the available evidence.

Schoolchildren who suffer from subclinical insomnia have a 28.0% higher prevalence of depressive symptomatology. This is similar to that reported by Shuang-Jiang et al. in their study that included Chinese adolescents and young adults aged 12 to 29 during part of the COVID-19 pandemic, finding in binary logistic regression analysis that depression is significantly associated as a risk factor for insomnia [93]. Likewise, Chunliu Luo et al. found that new incidence and persistence of insomnia was significantly associated with depression [94]. Our association could be due to the COVID-19 pandemic due to the higher incidence of depression leading to episodes of insomnia; because the risk of developing a new episode of major depression was higher in patients with insomnia at the beginning of the study and that persisted one year later. Physiologically, this can be explained by polysomnographic studies that are carried out in patients with episodes of major depression and present three types of anomalies: alteration of the continuity of sleep, reduction of deep N-REM sleep, especially in the first cycle of the night, and REM sleep abnormalities [95].

The female sex was associated with a higher prevalence of depressive symptomatology. This is similar to what was reported by Zeng Zhang et al. in three high schools, finding a positive association between sex and depression [70]. However, it differs from what was found by Ostovar S. et al., who concluded that there was a higher IAD with depression associated with males [25]. Our association is justified because, in adolescence, women are more likely to present depressive symptomatology due to biological and hormonal changes in the menstrual stage, due to hormonal fluctuations such as estrogen, which alter brain chemistry [96]. In addition, previous studies mention that female adolescents tend to feel bad for much longer than male adolescents, suggesting that they are more likely to suffer from depression [97], In addition to female adolescents, they need more approval and feel successful to be safe [98], among other factors such as sociocultural (greater willingness and ease of women to report and admit depressive and anxious feelings and the multiplicity of roles in the family) and biological (genetic predisposition, sex hormones, endocrine reactivity to stress, neurotransmission systems and neuropsychological determinants) [99].

### Factors associated with anxiety

The female sex was associated with a higher prevalence of anxiety. This is similar to what was reported in China, where there was a positive association between the female sex and anxiety [70]. However, it differs from what was found by Ostovar S. et al. in their research with 1052 adolescents revealed that men have higher mean scores in depression [25]. Our association is due to the differences between both sexes regarding the etiology and course of psychiatric disorders such as anxiety, since sexual differences have been evidenced, both in structure and function; limbic and anterior brain regions are extremely sensitive to hormones released during stress, especially glucocorticoids [100]. While the person's way of coping, their role based on gender, economic level, educational level, marital status, social support, social isolation, adversities during childhood, social changes, cultural norms and vulnerability to exposure to events stressors cause the prevalence of anxiety to vary according to sex [101].

Having an eating disorder (ED) increases the prevalence of anxiety by 37.0%. Being higher than that reported by Mérida C. et al. in an adolescent school in La Paz, where 86.0% of the ED Risk Group, 72.2% with anorexia and 88.2% with Bulimia present anxiety respectively. In addition, it demonstrates that there is a positive correlation between EDs and Anxiety [90]. However, bidirectional causality has been shown between the variables [102].

Having subclinical insomnia increases the prevalence of anxiety by 42.0%. This is similar to what was reported by Chunliu Luo et al. obtaining a positive correlation between insomnia and anxiety [94]. This association can be explained because the lack of sleep is established; the organism, through a physiological reaction, increases anxiety and stress levels, which generates greater alertness and difficulty to relax and rest [103]. The mechanisms associated with insomnia are a hormonal and neuronal imbalance that produces alterations in the secretion of cortisol, which suppresses sleep, among other hormones involved in the sleep–wake cycle [104].

Schoolchildren who reported using the Internet to interact socially increased the prevalence of anxiety by 31.0%. This is similar to what was said by Huanca J. in pre-university students from the city of Arequipa-Peru, finding that in the groups with the greatest number of hours connected to social networks they present a higher prevalence of anxiety [105]. Our association can be explained because adolescents report that their feelings of anxiety increase when they see their friends constantly on vacation or enjoying the nights; this can make young people feel that they are missing things while others enjoy life. These feelings can promote a "compare" and "desperation" effect. In conclusion, the often unrealistic images offered on social media can cause young people to have feelings of self-consciousness, low self-esteem, and the pursuit of perfectionism that can manifest as anxiety disorders [83].

### Implications of findings in mental health

Internet use has increased in the context of the COVID-19 pandemic. Our results provide information on a population that is little explored in our country, such as schoolchildren, who represent a vulnerable population in the face of stressful events. Implementing Internet access as a pillar in education has been transcendental for the continuity of education. However, its excessive use leads to the development of negative mental health symptoms through intermediary (associated) factors that must be taken into account in order to avoid their appearance through advisory programs focused on the correct use of electronic devices with the support of their parents as counselors, in addition to implementing semester-long training for all students where the complications of their excessive use are highlighted, taking into account that this age group is highly vulnerable [106].

### Strengths

To the authors' knowledge, this study is the first to study the prevalence of IA and its association in high school students. of a Peruvian region in this unprecedented context with a large population reached. Likewise, the questionnaires used are validated and reliable instruments to evaluate the study variables. The information obtained from the questionnaires was favorable thanks to the participatory will of the parents of the students and being a large population, they were completed adequately. Finally, the results of this research will serve as the basis for other studies on IAD and mental health.

### Limitations

A limitation of our study is the cross-sectional design, which did not allow the identification of causal relationships between the study variables. Likewise, the study could present biases due to lack of representativeness and because the non-probabilistic sample chosen is not an adequate reproduction to extrapolate the results to other regions of the country. The prevalence of IAD in our study may be underestimated because participation was voluntary and only based on the sincerity of the students. It could also be overestimated due to the greater interest of the participants who presented the symptoms. Another limitation is that confounding variables were not evaluated, such as the level of stress, the level of satisfaction with family communication [107], the duration of sleep, study and the amount of homework assigned [108], the educational level of the parents and number of members in the household [109] and family dysfunction [110] variables that were associated with the development of these negative mental health symptoms.

## Conclusions

We found that 2, 1, and 3 out of 10 students presented IAD, depressive symptomatology, and anxiety, respectively. We did not find an association between IAD and depressive symptomatology, but we did find an association with anxiety. Among the factors associated with the development of depressive symptomatology were the male sex, the presence of eating disorders, subclinical insomnia, using devices for more than 2 h, and using the Internet for academic activities. In relation to anxiety, the associated factors are the female sex, the presence of eating disorders, subclinical insomnia, and the use of the Internet as a social interaction. We recommend implementing counseling programs in view of the imminent introduction of the Internet as a pillar in education. 

## Data Availability

The dataset generated and analysed during the current study is not publicly available due data sharing restriction stablished by the ethics committee but are available from the corresponding author on reasonable request.
